# Outcome of patients with peritoneal metastasis from ovarian cancer treated with Pressurized IntraPeritoneal Aerosol Chemotherapy (PIPAC)

**DOI:** 10.1515/pp-2023-0049

**Published:** 2024-06-10

**Authors:** Ingrid Terese Foslund, Sahra Aisha Vinholt von Magius, Alan Patrick Ainsworth, Sönke Detlefsen, Claus Wilki Fristrup, Anja Oer Knudsen, Michael Bau Mortensen, Line Schmidt Tarpgaard, Kirsten Marie Jochumsen, Martin Graversen

**Affiliations:** Odense PIPAC Centre, 11286 Odense University Hospital, Odense, Denmark; Department of Obstetrics and Gynaecology, 11286 Odense University Hospital, Odense, Denmark; Department of Surgery, 11286 Odense University Hospital, Odense, Denmark; Department of Clinical Research, Faculty of Health Sciences, University of Southern Denmark, Odense, Denmark; Department of Pathology, 11286 Odense University Hospital, Odense, Denmark; Department of Oncology, 11286 Odense University Hospital, Odense, Denmark; OPEN – Open Patient Data Explorative Network, 11286 Odense University Hospital, Odense, Denmark

**Keywords:** Ovarian Cancer, Peritoneal Metastasis, Pressurized IntraPeritoneal Aerosol Chemotherapy (PIPAC), Peritoneal Regression Grading Score (PRGS), Quality of Life (QoL)

## Abstract

**Objectives:**

There are few data on Pressurized IntraPeritoneal Aerosol Chemotherapy with cisplatin and doxorubicin (PIPAC C/D) in women with primary unresectable or recurrent platinum-resistant peritoneal metastasis (PM) from ovarian cancer (OC). We evaluated survival, histological and cytological response, Quality of Life (QoL) and toxicity after PIPAC C/D in these patients.

**Methods:**

Retrospective analysis of patients from the prospective PIPAC-OPC1 and -OPC2 studies. The histological response was evaluated by the Peritoneal Regression Grading Score (PRGS). QoL questionnaires were collected at baseline and after third PIPAC or 60 days. Adverse events were collected until 30 days after the last PIPAC. Demographic and survival data were analysed based on intention to treat. Response, QoL and toxicity were analysed per protocol (≥1 PIPAC).

**Results:**

Twenty-nine patients were included. Five patients (17 %) were non-accessible at PIPAC 1. One patient was excluded due to liver metastases at PIPAC 1. Thus, 23 patients had 76 PIPACs (median 2, range 1–12). Median overall survival was 8.2 months (95 % CI 4.4–10.3) from PIPAC 1. Biopsy data were available for 22 patients, and seven (32 %) patients had a major/complete histological response (PRGS≤2) at PIPAC 3. No cytological conversions were registered. Symptoms and function scores worsened, while emotional scores improved. Three patients had severe adverse reactions (two ileus, one pulmonary embolism); no life-threatening reactions or treatment-related mortality was observed.

**Conclusions:**

PIPAC C/D was feasible and induced histological regression in a substantial proportion of patients with platinum-resistant PM from OC. Larger studies are needed to evaluate impact on survival.

## Introduction

Ovarian cancer (OC) is the eighth most common malignancy and the fifth most common cause of cancer mortality in women worldwide [1], [2], [3]. According to the Danish cancer registry, the age-adjusted incidence rate is 14 per 100,000 women, including tubarian, ovarian and primary peritoneal cancer [4]. More than 90 % have malignant epithelial type dominated by high-grade serous adenocarcinoma. Eighty percent are diagnosed in International Federation of Gynaecology and Obstetrics (FIGO)-stage III–IV, where the 5-year survival is only 25–36 % [5, 6]. Despite complete primary or interval debulking surgery including platinum- and taxane-based chemotherapy, most patients suffer from recurrent disease [7], [8], [9]. These patients may benefit from reintroduced platinum- and taxane-based chemotherapy with a median survival (mOS) of 30 months [10, 11]. Still, there is no standard treatment in patients with platinum-resistant OC, even if randomized controlled trials in selected patients show a median survival of 9–12 months after treatment with single agent chemo- or immunotherapy [12, 13].

Pressurized IntraPeritoneal Aerosol Chemotherapy (PIPAC) with cisplatin and doxorubicin (C/D) was introduced a decade ago as an intraperitoneal drug delivery system in patients with peritoneal metastasis (PM) [14, 15]. Tempfer et al. showed a histology-based response rate of 76 % and a mOS of 14.1 months after the first PIPAC C/D in 50 women with recurrent platinum-resistant OC with isolated PM [16]. The feasibility and safety of PIPAC has been shown in patients with PM from various primary tumours, and the procedure may be performed in the outpatient clinic [17, 18]. In a randomized controlled trial, Somashekhar et al. is currently investigating efficacy in 100 women with recurrent platinum-resistant ovarian cancer randomly allocated to PIPAC C/D or systemic chemotherapy [19]. Further, the PARROT trial showed that PIPAC C/D was feasible and showed a clinical benefit rate in 33/40 (82 %) women with recurrent platinum-resistant ovarian cancer [20]. Still, more treatment specific data on PIPAC in women with primary unresectable or recurrent platinum resistant OC are needed before evaluating efficacy in large randomized clinical trials.

Based on data from two prospective trials, this study aimed to report survival, histological/cytological response, Quality of Life (QoL) and toxicity in patients with PM from primary unresectable or recurrent platinum-resistant tubarian, ovarian or primary peritoneal high-grade serous adenocarcinoma.

## Materials and methods

This is a retrospective subgroup analysis of patients with primary unresectable or recurrent platinum-resistant PM from OC, who were included in the prospective PIPAC-OPC1 and -OPC2 trials at Odense PIPAC Centre, Denmark from 2015 to 2022. The inclusion and exclusion criteria have been described previously [21, 22]. These criteria were identical apart from the acceptance of Eastern Cooperative Oncology Group (ECOG) performance score (PS) 0–2 in PIPAC-OPC1, whereas only patients with PS 0–1 were accepted in PIPAC-OPC2. Further, patients with isolated PM were included in PIPAC-OPC1, while one extraperitoneal metastasis was allowed in PIPAC-OPC2.

Patients were discussed at a dedicated multidisciplinary tumour (MDT) conference prior to inclusion. If eligible, they were scheduled for a series of three PIPAC C/Ds at an interval of 4 to 6 weeks. The response to treatment was evaluated by histology of peritoneal quadrant biopsies (QBs), peritoneal lavage or ascites cytology, a computed tomography (CT) and onset or disappearance of symptoms. Patients were again discussed at the MDT conference and continued PIPAC if these endpoints did not lead to a conclusion of disease progression.

### Pressurized IntraPeritoneal Aerosol Chemotherapy (PIPAC)

The PIPAC C/D procedure has been described previously [15, 21]. In selected cases, patients were treated with electrostatic precipitation PIPAC (ePIPAC) according to an approved amendment to the PIPAC-OPC2 study [23].

### Outcomes

#### Survival

Survival was calculated from the date of PIPAC 1 in the intention to treat population.

#### Histological/cytological response

The QBs were evaluated according to the four-tiered Peritoneal Regression Grading Score (PRGS) [24]. In short, PRGS 1 denotes a complete histological response, PRGS 2 a major histological response, PRGS 3 a minor histological response and PRGS 4 no histological response. The PRGS was reported separately for each QB and as a mean value, for all biopsies from a given QB set. Up-front immunohistochemical staining for the epithelial cell adhesion molecule (EpCAM), a marker with high sensitivity and specificity for PM, was used in addition to conventional H&E staining of the biopsies, as described previously [21, 24], [25], [26], [27], [28], [29], [30]. The mean PRGS was calculated as the average of all QBs obtained prior to a given PIPAC C/D. A histology-based response to PIPAC was defined as a mean PRGS≤2 at PIPAC 3 (unless PRGS≤2 already at PIPAC 1) or an absolute mean PRGS reduction ≥1.0 from PIPAC 1 to PIPAC 3 [22, 26]. Peritoneal lavage fluid was aided by immunocytochemical staining on demand and graded as malignant cells, cells suspicious of malignancy, atypical cells, no malignant cells and other. A cytological response/progression was defined as conversion from malignant or suspicious cells to non-malignant cells and vice versa. Histological and cytological analyses were performed by the same pathologist to avoid inter-observer variability.

#### Quality of life

The European Organization for Research and Treatment of Cancer Quality of Life Questionnaire (EORTC QLQ-C30) was collected at baseline and after 60 days (OPC1 study) or after the third PIPAC (OPC2 study) [31].

#### Toxicity

A study nurse collected data on 30 days adverse events (Common Terminology Criteria for Adverse Events (CTCAE) version 4.0) and surgical complications according to the Clavien–Dindo classification [32, 33]. The causality to treatment was evaluated by the principal investigator and sponsor. Adverse events that were probably or certainly related to treatment were reported. To avoid double registration, surgical complications were exclusively defined as postoperative bleeding, intra-abdominal abscess or bowel perforation.

### Statistics

Patients assigned to PIPAC were defined as the intention to treat (ITT) population, and baseline characteristics and survival data were analysed according to ITT. Patients who completed ≥1 PIPAC were included in the per protocol (PP) population. Treatment-related data including response, QoL and toxicity were analysed PP. Values were given as means or medians where appropriate. Categorical data were specified with 95 % confidence intervals (95 % CI), and comparisons were performed using parametric or non-parametric tests after test for normal distribution. p-Values were two-tailed, and a p-value of 0.05 was considered statistically significant. Survival was calculated from date of PIPAC 1 and modelled in Kaplan–Meier survival curves.

### Ethical clearance

This study is a subgroup analysis of patients who consented to the prospective PIPAC-OPC1 and -OPC2 studies [21, 22]. These studies were conducted according to the Helsinki declaration.

## Results

**Figure 1:**
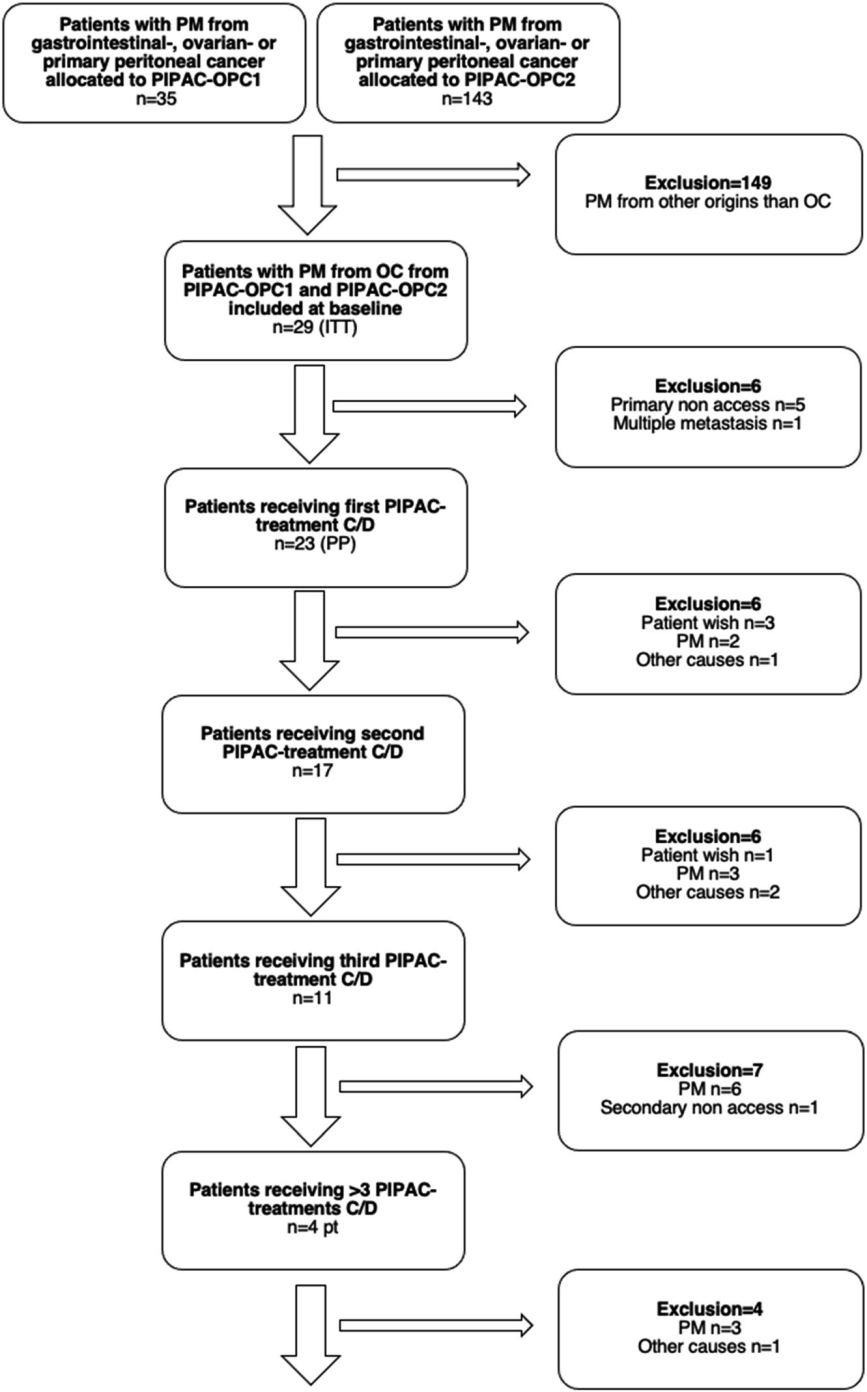
Flow chart of patient enrolment. C/D, cisplatin and doxorubicin; ITT, intention to treat; n, number of patients; OC, ovarian cancer; OPC, Odense PIPAC Center; PIPAC, Pressurized IntraPeritoneal Aerosol Chemotherapy; PM, peritoneal metastasis.

The PIPAC-OPC1 trial included 35 patients from March 2015 to October 2016, and the OPC2 trial (including the amendment on ePIPAC) included 143 patients from December 2016 to January 2022. Of these, 29 OC patients were eligible for the ITT analysis (Table 1 and Figure 1). The median (range) time from OC diagnosis to PIPAC 1 was 30 (3–131) months. Nineteen patients (66 %) had recurrence after complete or optimal debulking surgery. More than 1/3 of the patients had disease progression after first line single agent chemotherapy instituted after platinum resistancy, and none of the patients received synchronous systemic chemotherapy during or in between PIPAC. Three patients had extraperitoneal metastases at inclusion (two had supradiaphragmatic lymph node metastasis, one had lung metastasis). Five patients were excluded due to primary laparoscopic non access, and one was excluded due to the presence of liver metastases detected during standard laparoscopic ultrasound immediately prior to the PIPAC procedure. Ultimately, 23 patients (5 from PIPAC-OPC1 and 18 from PIPAC-OPC2) were included in the PP population, who had a total of 76 treatments (63 PIPACs and 13 ePIPACs, median 2, range 1–12).

**Table 1:** Baseline characteristics of ovarian cancer patients treated with Pressurized IntraPeritoneal Aerosol Chemotherapy with cisplatin and doxorubicin.

Number of included patients	29
Age, median years (range)	64 (42–77)
ECOG performance status 0, n (%)	8 (27.6)
ECOG performance status 1, n (%)	21 (72.4)
Primary unresectable ovarian cancer, n (%)	10 (34)
Platinum-resistant recurrent ovarian cancer, n (%)	19 (66)
Extraperitoneal metastasis at inclusion n (%)	3 (13)

**Previous treatment**	

Previous palliative chemotherapy, n (%)	29 (100)
Median lines of palliative chemotherapy (range)	2 (1–10)
One line	8 (28)
Two lines	10 (34)
>Two lines	11 (38)

ECOG, Eastern Cooperative Oncology Group; PIPAC, Pressurized IntraPeritoneal Aerosol Chemotherapy.

The mean (SD) PIPAC procedure time was 86 (20) minutes, and 42 (55 %) treatments were performed in the outpatient clinic. The median (range) follow-up from PIPAC 1 was 18 (3–52) months.

### Survival

**Figure 2:**
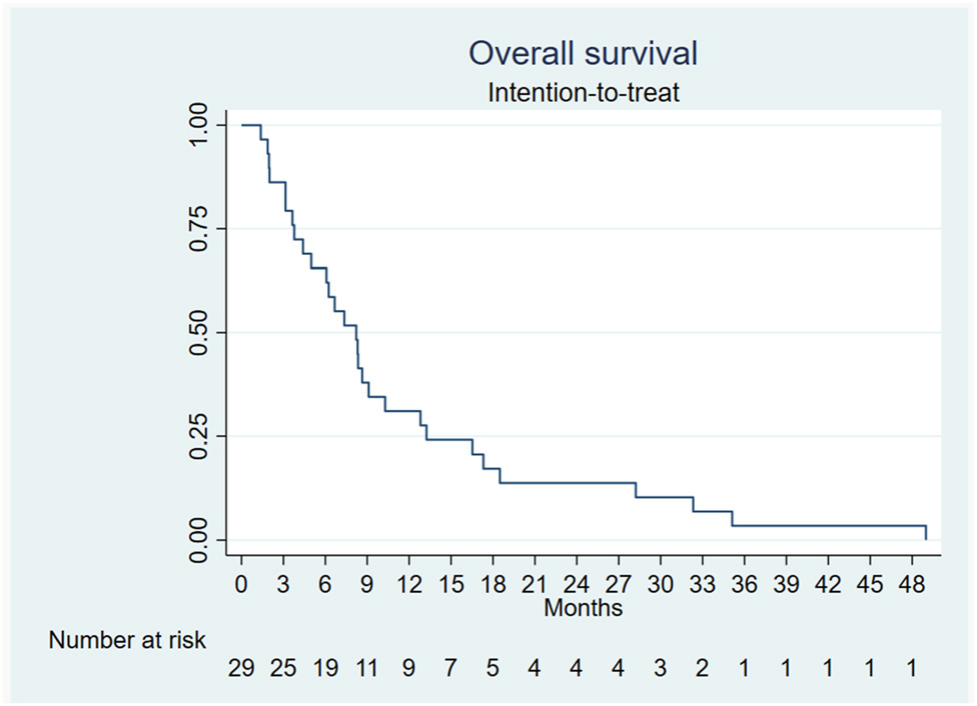
Overall survival after Pressurized IntraPeritoneal Aerosol Chemotherapy with cisplatin and doxorubicin.

The mOS was 8.2 months (95 % CI 4.4–10.3) after PIPAC 1 (ITT) (Figure 2).

### Histological/cytological response

Biopsy data were available in 22 patients. Two patients had a PRGS ≤2 at PIPAC 1, while 7/22 (32 %) patients had a major or complete histological response (mean PRGS≤2) at PIPAC 3, whereas no cytological conversions were registered (Table 2).

**Table 2:** Response evaluation of patients treated with Pressurized IntraPeritoneal Aerosol Chemotherapy with cisplatin and doxorubicin.

Histology			
	PIPAC 1	PIPAC 2	PIPAC 3
Number of patients with biopsy data	n=22	n=15	n=10

PRGS MEAN (SD)	2.86 (0.76)	2.19 (0.67)	1.98 (0.68)
MEAN PRGS 1 OR 2, n	2	7	7
PRGS MAX (SD)	3.22 (0.75)	2.60 (0.74)	2.40 (0.84)
MAX PRGS 1 OR 2, n	2	6	4

**Cytology**			

Number of patients with cytology data	n=21	n=15	n=10

Positive cytology	19	13	10
Cytological conversion	–	0	0

MAX, maximum; n, number of patients; PIPAC, Pressurized IntraPeritoneal Aerosol Chemotherapy; PRGS, Peritoneal Regression Grading Score; SD, standard deviation.

### Quality of life

**Figure 3:**
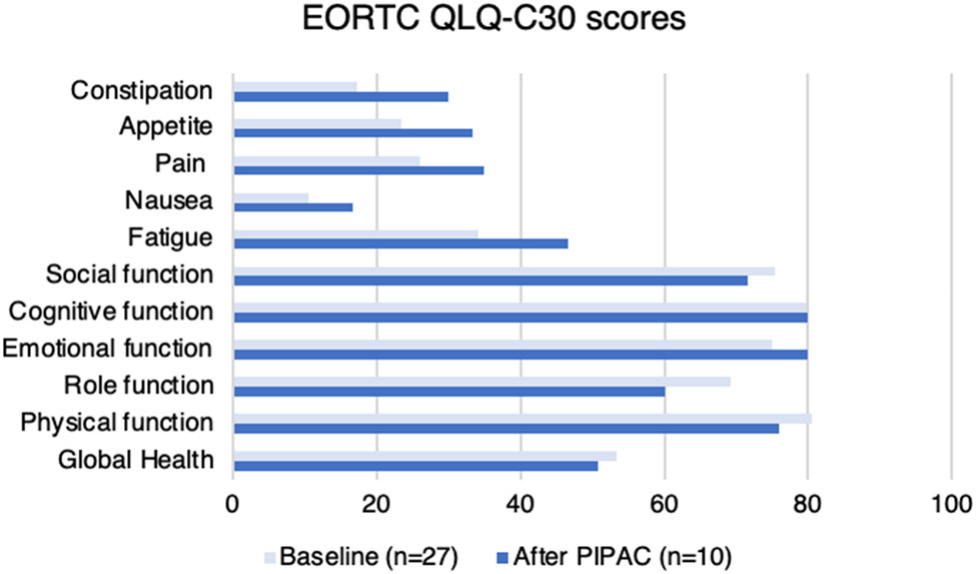
Quality of Life scores at baseline and after Pressurized IntraPeritoneal Aerosol Chemotherapy with cisplatin and doxorubicin. EORTC QLQ-C30, The European Organisation for Research and Treatment of Cancer Quality of Life Questionnaire 30; n, number of patients; PIPAC, Pressurized IntraPeritoneal Aerosol Chemotherapy.

Twenty-seven (including all data from ITT population) and 10 questionnaires were collected at baseline and after treatment, respectively (Figure 3). Overall, patients reported more symptoms and decreasing function scores apart from improved emotional and unchanged cognitive function.

### Toxicity

There was no treatment-related mortality and no life-threatening adverse reactions (Table 3). Three severe adverse reactions were recorded of which two patients had ileus (treated conservatively) and one patient had a pulmonary embolism. Pain, nausea and vomiting were the most common mild or moderate adverse reactions. No severe surgical complications were recorded, but we observed one case of wound dehiscence, one case of haematoma and three cases of fluid leakage from the port site scars.

**Table 3:** Procedure related data.

Procedure related data									
Number of PIPAC treatments, median (range)					76, 2 (1–12)				
Treatments performed with ePIPAC n (%)					13 (17)				
Outpatient procedures, n (%)					42 (55)				

**Adverse reactions**									

**Grade**	**Pain**	**Obstipation**	**Diarrhoea**	**Ileus**	**Nausea**	**Vomiting**	**Urinary retention**	**Wound infection**	**Other** ^**a**^

1–2 n	43	15	4	0	33	21	6	4	11
3 n	0	0	0	2	0	0	0	0	1
4 n	0	0	0	0	0	0	0	0	0
30-day mortality	0	0	0	0	0	0	0	0	0

**Surgical complications**									

**Grade**		**Bleeding**		**Abscess**		**Perforation**		**Other**	

1-2 n		1		0		0		5	
≥3a n		0		0		0		0	

PIPAC, Pressurized IntraPeritoneal Aerosol Chemotherapy; SD, standard deviation. ^a^Grade 1–2: one case of pneumonia, 10 cases of fatigue and sweating and one case of pulmonary embolism (grade 3).

## Discussion

This retrospective analysis of prospectively collected data from 29 patients with primary unresectable or recurrent platinum-resistant PM from OC showed a mOS of 8.2 months from PIPAC 1. It also showed a major or complete histological response (PRGS≤2) in 32 % of the patients at PIPAC 3 but no peritoneal cytology-based response. The QoL scores deteriorated slightly after PIPAC even though it was well tolerated with minimal toxicity.

We excluded five patients from the ITT population due to a non-accessible abdomen and one patient due to liver metastases at the index laparoscopic ultrasound. We, therefore, had a non-access rate of 17 %, which is in agreement with a recent review in which the primary and secondary non-access rates were between 0 and 17 % [18]. The non-access rate is also in agreement with Tempfer et al. and is arguably caused by the high rate of patients with adhesions due to previous primary or interval debulking surgery [16].

Survival of 8 months after PIPAC 1 is interesting in this group of patients with platinum-resistant disease. Tempfer et al. showed an even better mean survival of 331 days after PIPAC 1 in a prospective study of 53 women with PM from recurrent, platinum-resistant ovarian, tubarian or primary peritoneal cancer [16]. The difference in survival rates might be due to differences of the populations studied. One third of the patients in the present study were never resected, whereas all patients in the study by Tempfer et al. were amenable for primary/interval debulking. The AURELIA study showed a mOS of 16.6 months in platinum-resistant OC patients treated by a combination of chemotherapy and bevacizumab [34]. Importantly, the AURELIA study used strict inclusion criteria and, therefore, excluded patients who had more than two previous anticancer regimens or who progressed during platinum-based treatment, which hinders comparison of survival rates. The recent PARROT trial investigated feasibility and radiological response to PIPAC C/D in 43 women with platinum-resistant recurrent ovarian cancer [20]. The authors reported a survival of 27 months, which is impressive. This survival, however, was computed from the date of recurrence imposing obvious lead-time bias. Further, the PARROT trial only included patients with recurrent disease who had a maximum of two lines of systemic chemotherapy, whereas our study included 11 patients (38 %) who had more than two lines of chemotherapy prior to PIPAC.

Seven of ten patients who completed three PIPACs had a major or complete histological response to treatment, which is encouraging. In comparison, no patients in the aforementioned PARROT trial showed response according to PRGS. Of note, it is difficult to deduce the impact and clinical consequences of the biopsy strategy in the PARROT trial where biopsies were taken after administration of chemotherapy [20]. Most centres recommend biopsies before administration of chemotherapy [22, 35]. Data on the prognostic impact of PRGS are still dubious, and some studies have considered response to PIPAC as any decrease in PRGS during treatment [21]. Also, the biopsy strategy during response evaluation is not uniform, since some centres clips mark biopsy sites, while others biopsy new lesions at every PIPAC. Importantly, the recently published PIPAC-OPC2 study showed that a cut-off of mean PRGS≤2.0 or an absolute decrease of 1.0 from PIPAC 1 to 3 held positive prognostic value, which is also in accordance with the findings of Baake et al. [22, 36]. Further, the evaluation of PRGS is reproducible with a good to excellent inter-observer variability, and up-front immunohistochemistry can improve the reproducibility of the PRGS, particularly in less experienced observers [22, 25, 27, 37, 38].

We observed no cytology-based response from PIPAC 1 to 3. Cytology may be perceived as an adjunct to PRGS, especially regarding PM located to the visceral peritoneum, which is usually not biopsied. Still, the isolated sensitivity of conventional cytology is only 50–60 %, and studies have indicated that peritoneal cytology holds no independent prognostic value unless combined with the maximum PRGS score [24, 30, 39]. The examination of ascites or peritoneal lavage fluid may still play a crucial role, but perhaps more comprehensive molecular analyses are required to fully utilize its potential impact.

QoL evaluations were based on data retrieved at dissimilar time points after PIPAC 3 or 60 days, which impose an obvious selection bias as it excludes data from previous dropped out patients. The changes were based on only two different measurements of QoL in a small group of 10 women, so no firm conclusions could be drawn.

The adverse events profile was acceptable with mainly mild to moderate abdominal pain, nausea and vomiting. Two severe reactions of ileus were seen, but they were treated conservatively. The adverse reactions were thus manageable, which is in agreement with previous reports of complications and toxicity [21]. No severe surgical complications were observed.

Conclusions from the present study are limited by its retrospective design. Although data were collected in two prospective trials, they were not designed to be incorporated in this subgroup analysis. The inclusion criteria differed between studies, and some procedures (17 %) were completed with electrostatic precipitation, which might have altered the treatment efficacy. The PIPAC-OPC1 and -OPC2 studies accrued patients before the dose escalation study by Tempfer et al. in 2018 and, therefore, used cisplatin 7.5 mg/m^2^ and doxorubicin 1.5 mg/m^2^ [40]. From a methodological perspective, this should be considered a strength, but it might also preclude patients from a more effective treatment with higher doses of chemotherapy. No patients received bidirectional chemotherapy, which should be considered a strength, since it allowed the assessment of PIPAC monotherapy. On the other hand, this study does not provide feasibility and safety data of PIPAC in combination with systemic chemotherapy, which must be further investigated in similar study populations.

The efficacy of PIPAC C/D in patients with PM from OC must be examined further in randomized controlled trials. It could be of relevance to stratify OC patients into subgroups based on prior treatments, including both surgery and chemotherapy to evaluate which patients are most susceptible to PIPAC. Further studies must also evaluate the optimal drugs and doses for patients with OC treated with PIPAC. Perhaps the use of PIPAC with paclitaxel could be an interesting alternative but must await a recommended phase II dose, which is currently being investigated [41].

## Conclusions

In conclusion, PIPAC C/D was feasible and led to a major or complete histology-based response in a substantial proportion of patients with platinum-resistant OC. Randomized studies are warranted to show a potential survival benefit in patients with OC but also more prospective phase II studies that investigate the technical aspects of PIPAC such as optimal drugs, doses, pressure and diffusion time.
